# Oil biosynthesis in a basal angiosperm: transcriptome analysis of *Persea Americana* mesocarp

**DOI:** 10.1186/s12870-015-0586-2

**Published:** 2015-08-16

**Authors:** Aruna Kilaru, Xia Cao, Parker B. Dabbs, Ha-Jung Sung, Md. Mahbubur Rahman, Nicholas Thrower, Greg Zynda, Ram Podicheti, Enrique Ibarra-Laclette, Luis Herrera-Estrella, Keithanne Mockaitis, John B. Ohlrogge

**Affiliations:** Department of Biological Sciences, East Tennessee State University, Johnson City, TN 37614 USA; Department of Biomedical Sciences, East Tennessee State University, Johnson City, TN 37614 USA; Great Lakes Bioenergy Research Center, Michigan State University, East Lansing, MI 48824 USA; Bayer CropSciences, Morrisville, NC 27560 USA; School of Informatics and Computing, Indiana University, Bloomington, IN 47408 USA; Laboratorio Nacional de Genómica para la Biodiversidad-Langebio/Unidad de Genómica Avanzada UGA, Centro de Investigación y Estudios Avanzados del IPN, 36500 Irapuato, Guanajuato Mexico; Red de Estudios Moleculares Avanzados, Instituto de Ecología A.C., 91070 Xalapa, Veracruz Mexico; Department of Biology, Indiana University, Bloomington, IN 47405 USA; Department of Plant Biology, Michigan State University, East Lansing, MI 48824 USA

## Abstract

**Background:**

The mechanism by which plants synthesize and store high amounts of triacylglycerols (TAG) in tissues other than seeds is not well understood. The comprehension of controls for carbon partitioning and oil accumulation in nonseed tissues is essential to generate oil-rich biomass in perennial bioenergy crops. *Persea americana* (avocado), a basal angiosperm with unique features that are ancestral to most flowering plants, stores ~ 70 % TAG per dry weight in its mesocarp, a nonseed tissue. Transcriptome analyses of select pathways, from generation of pyruvate and leading up to TAG accumulation, in mesocarp tissues of avocado was conducted and compared with that of oil-rich monocot (oil palm) and dicot (rapeseed and castor) tissues to identify tissue- and species-specific regulation and biosynthesis of TAG in plants.

**Results:**

RNA-Seq analyses of select lipid metabolic pathways of avocado mesocarp revealed patterns similar to that of other oil-rich species. However, only some predominant orthologs of the fatty acid biosynthetic pathway genes in this basal angiosperm were similar to those of monocots and dicots. The accumulation of TAG, rich in oleic acid, was associated with higher transcript levels for a putative stearoyl-ACP desaturase and endoplasmic reticulum (ER)-associated acyl-CoA synthetases, during fruit development. Gene expression levels for enzymes involved in terminal steps to TAG biosynthesis in the ER further indicated that both acyl-CoA-dependent and -independent mechanisms might play a role in TAG assembly, depending on the developmental stage of the fruit. Furthermore, in addition to the expression of an ortholog of WRINKLED1 (WRI1), a regulator of fatty acid biosynthesis, high transcript levels for *WRI2-like* and *WRI3-like* suggest a role for additional transcription factors in nonseed oil accumulation. Plastid pyruvate necessary for fatty acid synthesis is likely driven by the upregulation of genes involved in glycolysis and transport of its intermediates. Together, a comparative transcriptome analyses for storage oil biosynthesis in diverse plants and tissues suggested that several distinct and conserved features in this basal angiosperm species might contribute towards its rich TAG content.

**Conclusions:**

Our work represents a comprehensive transcriptome resource for a basal angiosperm species and provides insight into their lipid metabolism in mesocarp tissues. Furthermore, comparison of the transcriptome of oil-rich mesocarp of avocado, with oil-rich seed and nonseed tissues of monocot and dicot species, revealed lipid gene orthologs that are highly conserved during evolution. The orthologs that are distinctively expressed in oil-rich mesocarp tissues of this basal angiosperm, such as WRI2, ER-associated acyl-CoA synthetases, and lipid-droplet associated proteins were also identified. This study provides a foundation for future investigations to increase oil-content and has implications for metabolic engineering to enhance storage oil content in nonseed tissues of diverse species.

**Electronic supplementary material:**

The online version of this article (doi:10.1186/s12870-015-0586-2) contains supplementary material, which is available to authorized users.

## Background

Basal angiosperms are the first and oldest families of flowering plants that originated well over 100 million years ago and are represented by only a few hundred species compared with hundreds of thousands of species of monocot and eudicot angiosperms [1, 2]. Avocado (*Persea americana*) belongs to the family *Lauraceae*, one of the largest basal angiosperm families with over 50 genera [3] and has been used extensively as a model system to understand the early evolution of angiosperm flower development from the gymnosperms [1, 4]. Avocado is also an advantageous system in which to study the evolution of mechanisms underlying the synthesis of storage reserves such as starch or lipids in fruit tissues other than seed. Interestingly, avocado fruit growth, unlike most angiosperm fruits, is characterized by an unrestricted period of cell division, which continues through the entire period of fruit development [5, 6]. During its development, the fleshy edible part accumulates by dry weight 60 to 70 % oil and 10 % carbohydrates. The oil is stored in the form of triacylglycerol (TAG) and is predominantly composed of oleic acid [7]. About 60 % of the total carbohydrates are seven-carbon sugar derivatives such as D-mannoheptulose and its sugar alcohol, perseitol [8]. The high nutritional value and the usefulness of avocado’s monounsaturated oils in promoting health raised its current world-wide production value to ~3.8 billion US dollars [9].

The avocado fruit, like oil palm and olive, is one of a few examples in which the mesocarp, a nonseed tissue, accumulates copious amounts of TAG. In general, TAG biosynthesis in plant tissues primarily involves synthesis of fatty acids in the plastid and their transfer to the endoplasmic reticulum (ER) followed by sequential esterification to a glycerol-3-phosphate backbone in an acyl-CoA-dependent [10] or -independent manner [11, 12]. Although biosynthesis of TAG in plants is generally understood and considered to be a highly conserved process, the molecular and biochemical details are mostly limited to oilseeds [13, 14]. Recently, greater attention is being given to plants that store oil in tissues other than seeds, which has revealed important differences [15–19]. For example, in avocado and oil palm mesocarp, lipid-droplet associated proteins (LDAP), which may play a role in stabilization of lipids, have been identified [20, 21]. Typically, storage proteins such as oleosins*,* caleosins, and steroleosins were shown to play a role in stabilization and regulation of the size of the oil bodies in angiosperm seeds and pollen [22]. However, several studies, including comparative transcriptome analysis of nonseed oil-rich tissues, consistently point to the absence or reduced transcript levels for genes encoding for these integral lipid-body proteins [15, 16, 23].

Transcriptome studies of oil palm and olive have also indicated key differences in the transcriptional control of TAG biosynthesis in nonseeds from that of seed tissues [15, 16, 18]. In seed tissues, many of the master regulators of embryogenesis and seed maturation, such LEAFY COTYLEDON (LEC) genes *LEC1*, *LEC1*-like (*L1L*), *LEC2* and FUSCA3 (*FUS3*), and abscisic acid (ABA)-insensitive3 (*ABI3*) regulate TAG synthesis directly or indirectly through the downstream transcription factor WRINKLED1 (*WRI1*; [24–28]). The WRI1 protein, a member of the APETALA2 (AP2)-ethylene responsive element binding proteins, regulates late glycolysis and fatty acid biosynthetic genes by binding to their promoter sequences [24, 29, 30]. Furthermore, along with WRI1, WRI3 and WRI4 were also shown to play a role in fatty acid biosynthetic pathway in floral and other nonseed tissues [31]. Interestingly, high transcript levels for homologs of WRI1, but not WRI3 and WRI4, were noted in coordination with oil accumulation in developing mesocarp of oil palm [16, 18, 32]. Successful complementation of *Atwri1* with *EgWRI1* further suggested that WRI1 is not only conserved between dicots and monocots but also regulates fatty acid biosynthesis in both seed and nonseed tissues [33].

While there has been major progress in our understanding of lipid biosynthesis in various plants and tissue types, gaps still remain with regard to how carbon partitioning is regulated and the oil content and composition is dictated [14, 16, 18, 27, 32–34]. Additional transcription factors that may play a role in controlling the enzymes, such as the acyltransferases, needed in later steps of TAG accumulation, also remain elusive. In this study we have asked which genes associated with lipid biosynthesis are predominantly expressed and how their expression patterns in the oil-rich mesocarp tissue of a basal angiosperm vary compared to those of monocot and dicot tissues. To address these questions and to further examine the evolutionary relationship of lipid biosynthesis genes across plants, we conducted quantitative analysis of RNA from developing mesocarp of avocado. Because of the distinctive position *P. americana* occupies in plant evolution it serves as an excellent system in which to probe conservation of regulatory mechanisms in lipid synthesis.

## Results and discussion

**Fig. 1:**
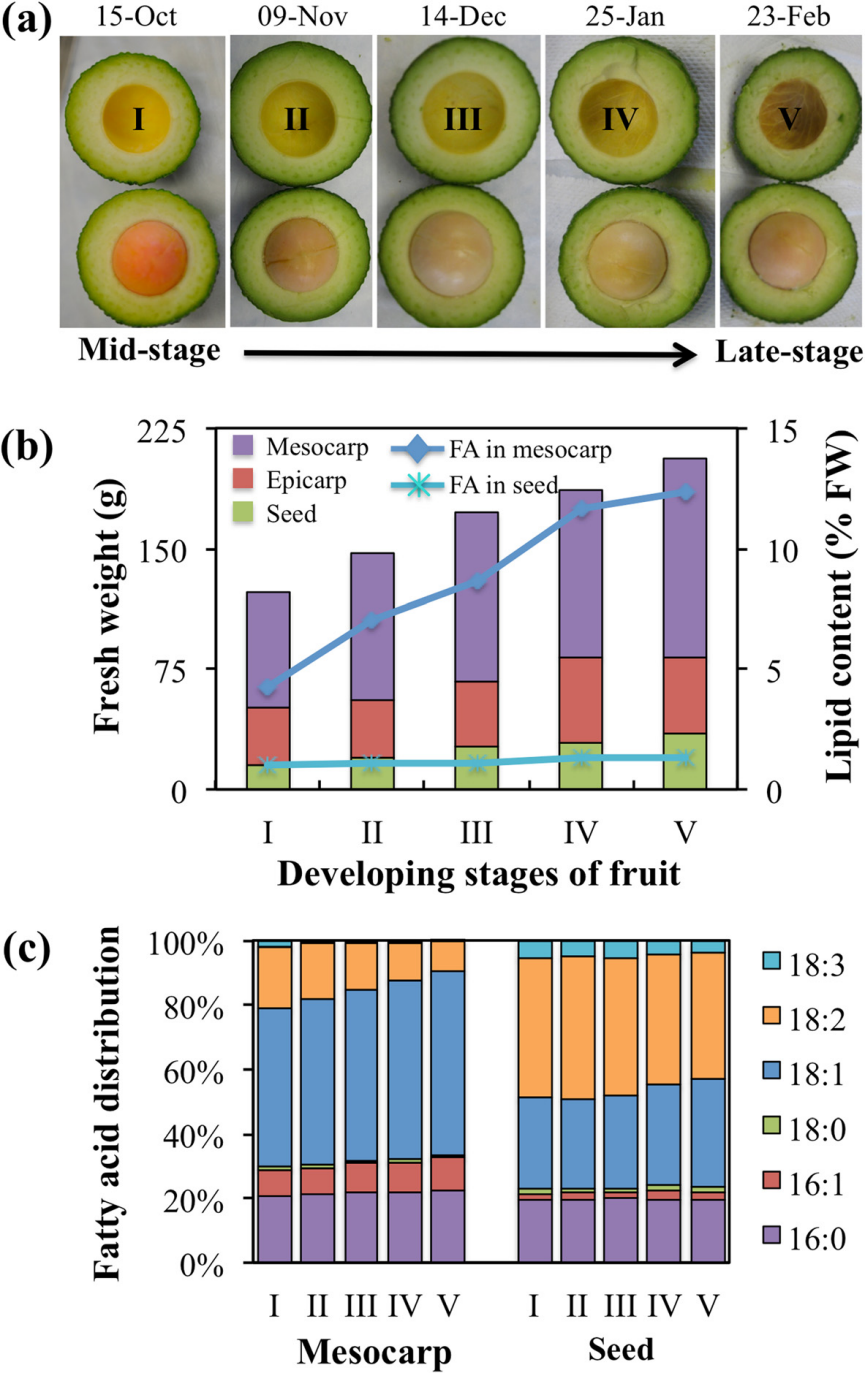
Lipid content and composition of developing fruits of avocado. **a** The five developing stages (I to V) of avocado fruits used for transcriptome analysis. **b** Fresh weight of various developing tissues with fatty acid (FA) content in mesocarp and seed. **c** Fatty acid composition of developing mesocarp and seed of avocado

Basal angiosperms, to which *P. americana* belongs, originated before the separation of monocots and dicots and contain features that are common to both groups. Transcriptome analysis of fatty acid biosynthesis in oil-rich nonseed fruit tissue has been previously reported for mesocarp of olive, a dicot [15] and oil palm, a monocot [16, 18]; similar studies of the more highly diverged basal angiosperms have not been reported. In this study, avocado mesocarp was selected for investigation of lipid biosynthesis in oil-rich tissue of an early angiosperm lineage. The mesocarp tissue from five stages of avocado fruits (I-V), with fresh weights ranging from ~ 125 to 200 g (Fig. 1a), was used to generate temporal transcriptome data, using next-generation sequencing methods (Additional file 1: Table S1). In order to associate expression patterns of lipid biosynthesis genes with temporal oil accumulation, the fatty acid content and composition of mesocarp was also analyzed (Fig. 1b and c). Details of the avocado RNA-Seq datasets available are summarized in Additional file 1: Table S1 and in NCBI BioProject PRJNA253536. Predicted functional annotation of contigs represented by at least 10 reads per kilobase per million mapped reads (RPKM) was based on BlastP alignment to lipid biosynthetic pathway proteins of *Arabidopsis thaliana* and is provided in Additional file 1: Table S2, along with the contig sequences (Additional file 2: Data S1). It must be noted that although transcript levels may not always reflect protein abundance or enzyme activity, similar transcriptome data has been successfully used previously to identify crucial steps in biochemical pathways [14, 16, 18]. Gene functional predictions most relevant to this study, along with their expression levels during mesocarp development are listed in Additional file 1: Table S3.

### Relationship of avocado mesocarp lipid accumulation with fruit growth

The fruit of avocado is a single-seeded berry and its development and growth lasts for more than nine months. Typically, early stage fruits, harvested at about 50 days after full bloom (DAFB) weigh ~ 10 g and their weight is increased by ten-fold when harvested at 88 DAFB and more than 20-fold by 230 DAFB [35]. The stage I ‘Hass’ fruits utilized in this study were harvested ~100 DAFB and weighed about 125 g, while the mature fruits in stage V reached an average weight of 230 g. The mesocarp of fruit contributed to about two-thirds of the total fruit weight and continued to increase with development (Fig. 1b). The increase in fruit weight was highly correlated with the accumulation of lipid content in the mesocarp tissue (R^2^ = 0.978; Additional file 3: Figure S1). The stage V fruits, with about 12 % oil by fresh weight, contained three-fold higher oil content, relative to stage I fruits (Fig. 1b). About one-fourth of the total oil content of the mesocarp was already accumulated in stage I fruits used in this study, which suggests that the lipid synthesis was initiated at an earlier stage of development. Based on the lipid content and fruit weight, the fruits harvested during October to February are estimated to represent mid to mature stages of fruit development (Fig. 1a). Interestingly, unlike mature oilseeds, mature ‘Hass’ avocados are capable of maintaining oil accumulation up to 18 % even after harvesting, until ripening [36]. In contrast to the mesocarp, avocado seed oil content was much lower and changed little throughout the development (Fig. 1b).

The fatty acid composition was tissue-specific and varied with development for mesocarp (Fig. 1c). Among the major fatty acids, oleic acid (18:1) was most abundant in mesocarp while in seeds linoleic acid was predominant throughout the development (Fig. 1c). The variation in mesocarp composition for 16:0, 16:1 and 18:0, during mid to late stage of development was small; a steady increase in 18:1 and concurrent decline in 18:2 proportion was notable (Fig. 1c). Seeds showed almost no variation in composition during the development and unlike in mesocarp, they contained a higher proportion of linolenic acid and lower 16:1 (Fig. 1c). Overall, the data indicate that the rate of mesocarp oil accumulation and changes in its composition were directly correlated with fruit development and increase in its biomass (Fig. 1 and Additional file 3: Figure S1). Fruit development and growth, including accumulation of its storage metabolites, are highly coordinated processes that are regulated by cross talk between various hormones. Several studies, indeed, have shown that exogenous ABA treatment enhances TAG accumulation by inducing the expression of various lipid biosynthesis genes as observed in developing seeds of *B. napus* [37, 38] and castor [39]. The hormone-mediated mechanisms by which fruit development and lipid accumulation are coordinated in avocado, however, remain to be elucidated.

### Transcript analysis of select lipid metabolic pathways of avocado mesocarp revealed patterns similar to that of other oil-rich species

**Fig. 2:**
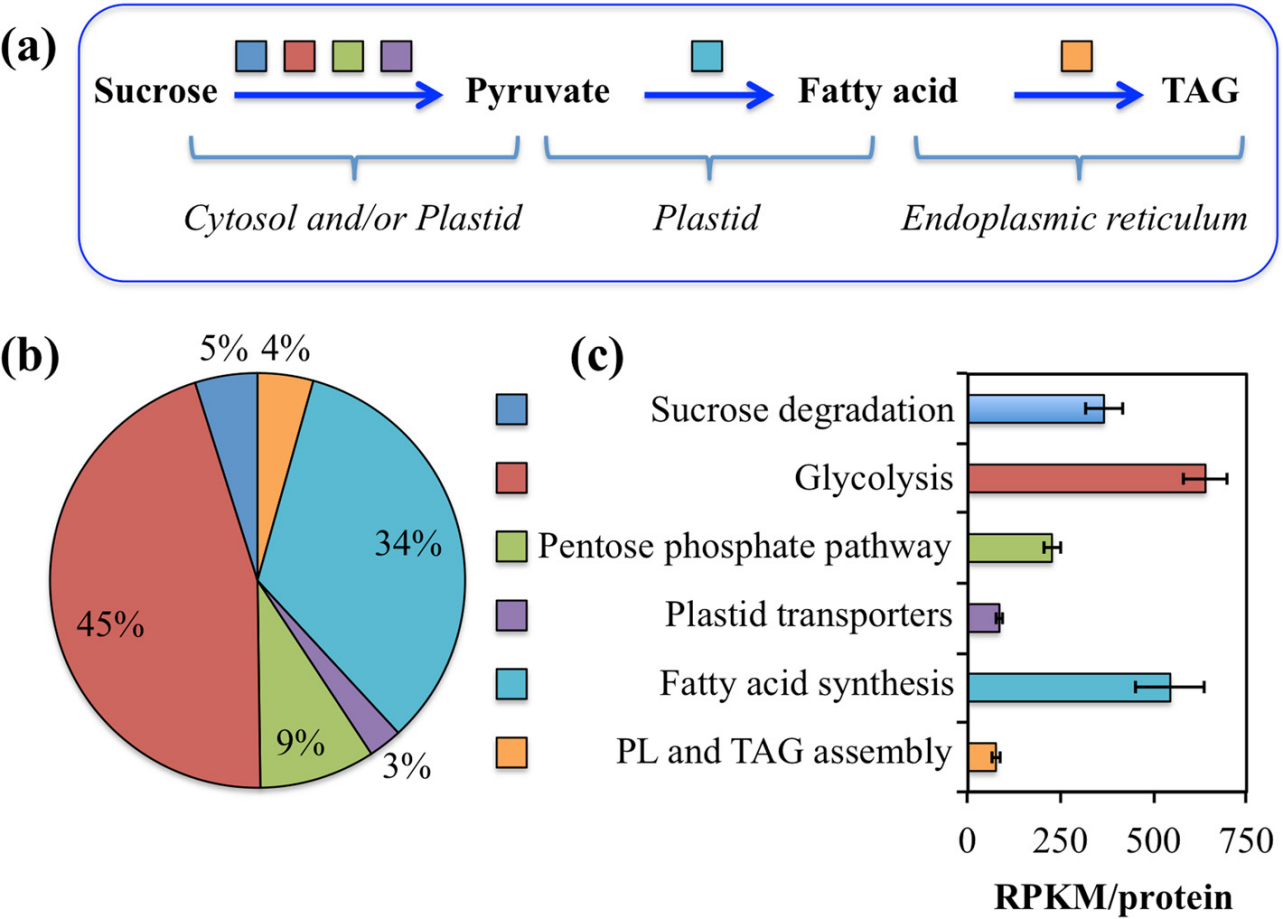
Gene expression pattern for select pathways (Additional file 1: Table S3). **a** Schematic of the pathways involved in conversion of sucrose to triacylglycerol (TAG). **b** The distribution of transcripts among the six pathways. **c** The number of reads per kilobase per million mapped reads (RPKM) per protein in each pathway. Multiple protein isoforms or subunits of a multi-protein complex were considered as a single protein and their transcripts were summed (Additional file 1: Table S3). The data are average transcript levels of five developing stages of mesocarp with error bars representing their standard deviation

The conversion of sucrose to TAG involves degradation of sucrose, generation of pyruvate in the plastid, which involves glycolysis, pentose phosphate pathway and plastid transporters, fatty acid synthesis in the plastid and TAG assembly in the ER (Fig. 2a). These six metabolic pathways require expression of over 200 genes (Additional file 1: Table S3). In avocado mesocarp, about 45 % of the transcripts corresponded to genes involved in glycolysis and 34 % to those in plastidial fatty acid biosynthesis (Fig. 2b). The analyses we undertook were designed to discover conserved functions in lipid biosynthesis and regulation in avocado, without regard to separation of close paralogs or allelic transcripts in the RNA datasets. Therefore, multiple transcripts encoding for genes of the same protein family or protein complex were summed and represented as RPKM/protein (Additional file 1: Table S3). More detailed analyses using whole genome assemblies will aid in further gene family member resolution. Overall, the average RPKM/protein, based on conserved protein annotation, across the five developmental stages of the mesocarp, were also abundant for those genes involved in glycolysis or the generation of pyruvate and subsequently fatty acid synthesis (Additional file 1: Table S3; Fig. 2c).

Notably, the high proportion and the high RPKM/protein of transcripts associated with acyl group synthesis in the plastid, was in contrast to the pattern observed for transcript levels for genes in phospholipid synthesis and TAG assembly (Fig. 2b). In fact their relative abundance remained the lowest among the six metabolic pathways that were analyzed and the transcript levels did not vary among developmental stages of the mesocarp (Additional file 1: Table S3; Fig. 2c). A similar contrast in the pattern of enhanced expression levels for genes involved in plastid fatty acid synthesis and comparatively minor changes in transcripts for most genes that participate in later steps of TAG assembly was also observed in oil-rich seed and nonseed tissues of dicots and monocots [14, 16]. These data suggest that a common enzyme stoichiometry and temporal regulation of transcripts associated with oil accumulation is conserved in different oil-rich tissues and in diverse species.

### Only some predominant orthologs of the fatty acid biosynthetic pathway in avocado are similar to that of monocots and dicots

**Fig. 3:**
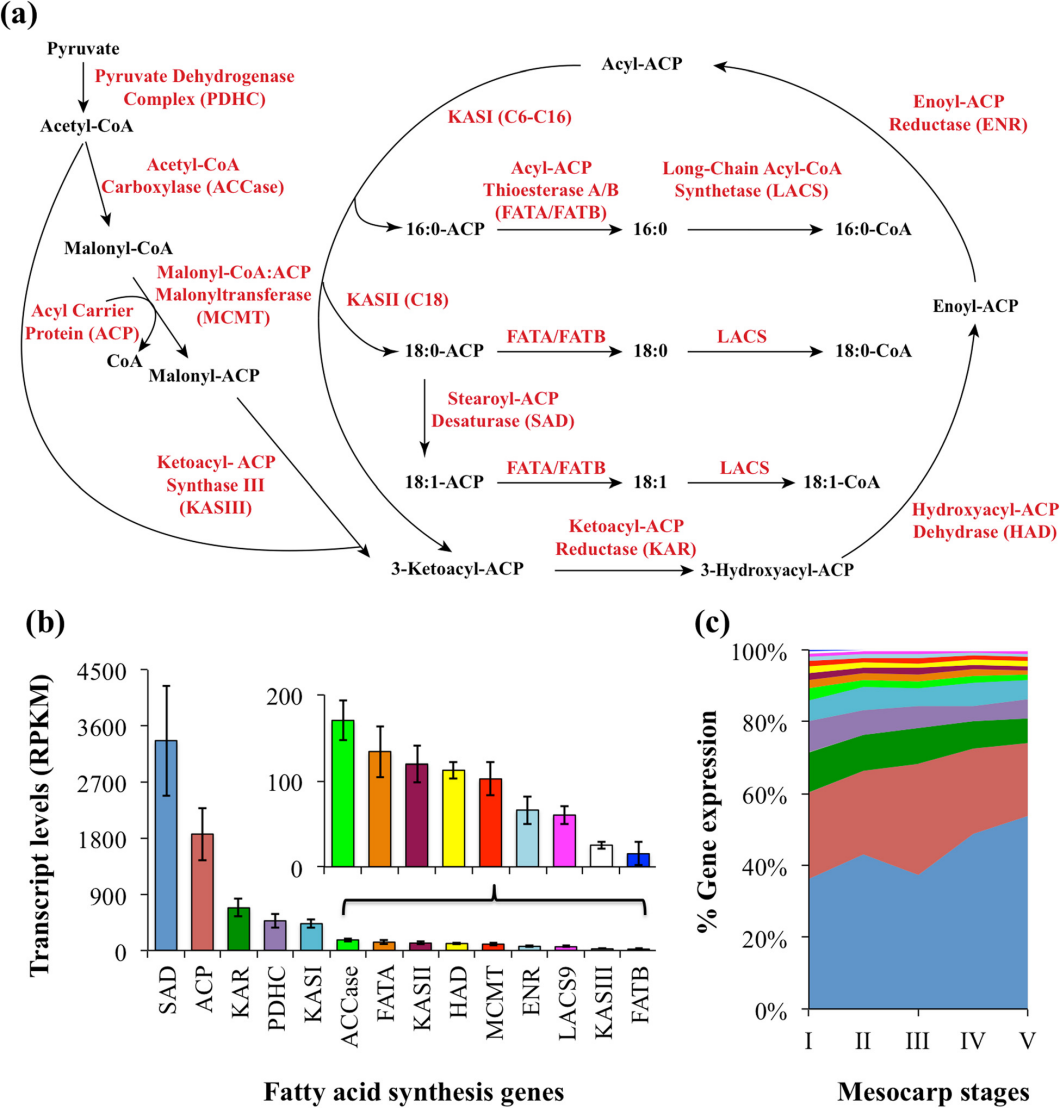
Expression levels for plastidial fatty acid synthesis genes. **a** Schematic of fatty acid synthesis pathway with protein names indicated in red color. **b** Transcript levels for each protein. **c** The relative distribution of transcript levels for each protein during mesocarp development. The data, reads per kilobase per million mapped reads (RPKM), are average transcript levels of five developing stages of mesocarp with error bars representing their standard deviation. The RPKM values for subunits of a protein and for multiple isoforms were summed (Additional file 1: Table S3)

**Fig. 4:**
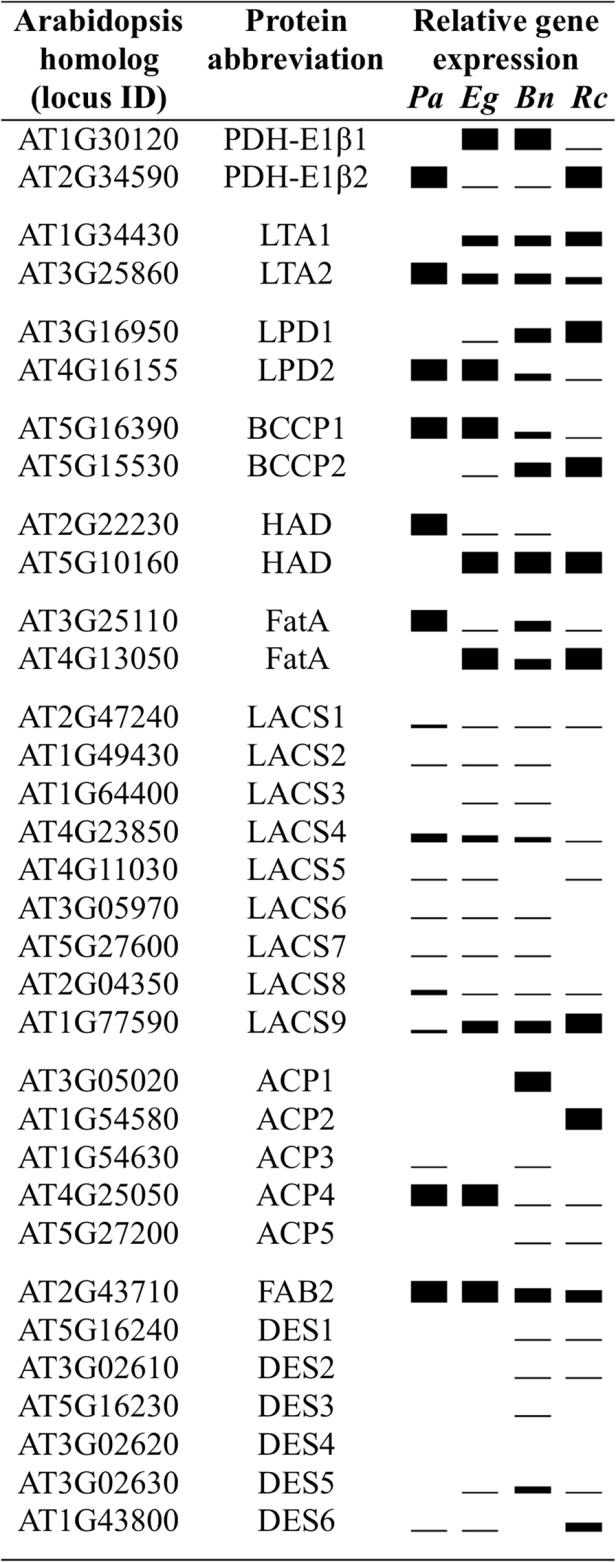
Relative gene expression levels for protein isoforms associated with fatty acid biosynthesis in oil-rich tissues of avocado (*Pa*), oil palm (*Eg*), rapeseed (*Bn*) and castor (*Rc*). Protein abbreviations are provided in Fig. 3a or Additional file 1: Table S3

The conversion of pyruvate to fatty acids in the plastid involves at least fourteen enzymes and/or protein complexes (Fig. 3a). Several of these proteins are encoded by more than one gene in *Arabidopsis* (Additional file 1: Table S3; [40, 41]. Comparison of the transcript levels of the orthologs of the gene family members in oil-rich tissues of avocado, oil palm, rapeseed and castor, while indicating some similarities across diverse species and tissues, also revealed several exceptions for avocado (Fig. 4). For example, among the three enzyme components of the pyruvate dehydrogenase complex (PDHC), while the E1α subunit of a heterodimeric protein (E1α_2_β_2_) is encoded by a single gene, the E1β subunit of E1 component, and E2 (dihydrolipoamide acetyltransferase, LTA), and E3 (dihydrolipoamide dehydrogenase, LPD) components are apparently encoded by two genes [42–44]. While the transcripts for orthologs of all the genes that encode for *Arabidopsis* PDHC components were detectable in oil-rich tissues of *B. napus*, oil palm and castor, only one ortholog for each of these components was detected in avocado (Fig. 4). The expression of a single ortholog in avocado was also noted for other enzymes that are typically encoded by more than one gene in angiosperms (Fig. 4). In the case of biotin carboxyl carrier protein (BCCP) of heteromeric acetyl-CoA carboxylase (ACCase), only the ortholog for BCCP1 (AT5G16390) was represented in the avocado mesocarp transcriptome. Both BCCP1 and 2 orthologs were detectable in oil palm, rapeseed and castor but BCCP1 was the predominant isoform in oil palm mesocarp, while BCCP2 was predominant in castor and rapeseed (Fig. 4). Similarly a single ortholog was represented in avocado transcriptome for hydroxyacyl-[acyl-carrier-protein (ACP)] dehydratase (HAD), and acyl-ACP thioesterase A (FATA), while both orthologs were at least detectable in the transcriptome of oil palm mesocarp and *B. napus* and castor seeds (Fig. 4). Furthermore, the ortholog that was expressed in avocado for PDHC-E1β, HAD, and FATA was different from the one that was predominantly expressed in oil-rich tissues of monocots and dicots (Fig. 4; [14, 16]. The absence of the second ortholog for PDHC-E1β, LPD, BCCP, and HAD genes in a basal angiosperm species was also observed at the genome level [17]. These data suggest that perhaps different/additional orthologs may have evolved to participate in fatty acid synthesis in seed and nonseed tissues of monocots or dicots, compared to a basal angiosperm.

More than 60 % of the transcripts encoding for fatty acid biosynthesis pathway proteins mapped to stearoyl-ACP desaturases (SAD/DES) and to ACP. In addition, their transcript levels increased with the maturity of the mesocarp (Fig. 3b and c), coinciding with the oil accumulation pattern (Fig. 2b). In arabidopsis, SAD/DES and ACP are encoded by seven and five member gene families, respectively, the largest gene families for any proteins in plastid fatty acid synthesis [40, 41, 45]. The ortholog for SAD that was expressed abundantly in oil-rich tissues was the same across all seed and nonseed tissues of diverse species that were compared (Fig. 4). In contrast, the major ortholog that was expressed for ACP, the cofactor that carries acyl-intermediates during fatty acid synthesis, varied across the species (Fig. 4). In avocado mesocarp, the expression levels of ACP transcripts represented about 24 % of the total fatty acid synthesis gene expression (Fig. 3a and b). Among the orthologs for the five ACP genes, transcripts that mapped to ACP4 (AT4G25050) were by far the most abundant in avocado; the other isoforms were either barely detectable or not represented (Fig. 4). Interestingly, while ACP4 ortholog transcripts were also abundant in oil palm [16], it was the least expressed or undetectable in embryos of rapeseed and nasturtium and embryo or endosperm of castor, where ACP1, ACP3, and ACP2, respectively, were predominant [14]. Previous studies have shown that multiple isoforms of ACP evolved early in plant evolution and that their expression is primarily dependent on the tissue type [46, 47] and differentially regulated, such as the light-responsive induction of ACP4 [48]. The abundance of the ACP4 ortholog in oil-rich mesocarp of both a basal angiosperm and a monocot fruit mesocarp suggests that ACP4 isoform might have evolved early to respond to demand for fatty acid biosynthesis for storage as TAG in photoheterotrophic nonseed tissues.

### Expression pattern of stearoyl-ACP desaturase genes in avocado reflects its lipid composition

During the development of avocado mesocarp, transcript levels for the ortholog of *Arabidopsis* SAD (AT2G43710; FAB2) were the most abundant than for any enzyme of lipid biosynthesis considered in this study, and constituted about 44 % of all the plastidial fatty acid synthesis gene expression (Fig. 3b and c). Although higher transcript levels for SAD in oil-rich tissues was not unexpected based on its very low catalytic turnover rate (0.5 s^−1^; [49, 50], it is noteworthy that in avocado, its levels were more than 100-fold higher relative to the expression levels for the ortholog of β-ketoacyl-ACP synthase III (KAS III; AT1G62640; Fig. 4 and Additional file 4: Figure S2). Similarly, *B. napus* embryo and endosperm of castor, which contain 30–90 % oleic acid or its derivatives, the transcript levels were more than 50-fold higher than KASIII (Additional file 4: Figure S2), correlating with their oil composition [14, 16]. The isoforms of SAD are responsible for introducing the first double bond into stearoyl-ACP to produce oleoyl-ACP (18:1^Δ9^-ACP). In contrast, oil palm mesocarp, which contains <40 % of monounsaturated fatty acids, the SAD transcript levels were only 16-fold higher than KASIII (Additional file 4: Figure S2). In date palm mesocarp, which is almost oil-free, transcripts for the orthologs of desaturases were only 3-fold higher than that of KASIII (Additional file 4: Figure S2). In *Arabidopsis*, *fab2* mutants showed reduced levels of 18:1 that were not restored by the other desaturase isoforms, except DES1 [51]. In avocado mesocarp, the transcript levels for the FAB2 ortholog were not only abundant but also increased with maturation (Figs. 3c, 4, Additional file 4: Figure S2) and correlated with increased 18:1 content (Fig. 1c), consistent with its role as a key determinant of the avocado oil composition.

### ER- rather than plastid-associated acyl-CoA synthetase transcripts are most highly expressed in avocado mesocarp

Long-chain acyl-CoA synthetases (LACS) participate in thioesterification of free fatty acids that is required for the utilization of fatty acids by most lipid metabolic enzymes. In *Arabidopsis* nine isoforms of LACS have been identified to participate in fatty acid and glycerolipid metabolism [52, 53]. In avocado mesocarp, transcripts for the ortholog of LACS4 were the most abundant, followed by LACS8, LACS1, and LACS9 (Fig. 4). These data were in contrast to the observations made in oil-rich seeds [14, 54] and nonseed tissues [16], where LACS9 transcripts were most abundantly expressed (Fig. 4). Plastid LACS9 was indeed considered as the major LACS isoform that is involved in the production of acyl-CoA for membrane glycerolipid and storage TAG synthesis in *Arabidopsis* [53] although transcripts for LACS8, LACS4, LACS2 and LACS1 were also found to be abundant in developing seeds of *Arabidopsis* [55]. Mutational studies in *Arabidopsis* revealed that TAG accumulation was not affected in loss-of-function *lacs8* and *lacs9* double mutant but the fatty acid levels reduced by 11 and 12 % in *lacs1* and *lacs9* double and *lacs1*, *lacs9*, and *lacs8* triple mutants respectively, which suggested possible overlapping roles of LACS1 and LACS9 [55]. In sunflower seeds, however, expression levels for the ortholog of LACS9 and LACS8 isoform were high during fatty acid synthesis and LACS8 has been considered as a candidate functioning similarly to LACS9 [54]. More recently, both LACS4 and LACS9 were shown to share an overlapping function in importing fatty acids from the ER to the plastid [56].

In avocado mesocarp, with more than 80 % of the transcripts of LACS orthologs represented by the ER-associated isoforms (LACS1, LACS4 and LACS8) and only 16 % contributed by the ortholog of plastidial LACS9 (Fig. 4), it remains unclear as to which of the LACS may contribute to acyl activation and where it may occur. Recently, FAX1 (At3g57280), a plastid localized protein was shown to mediate export of free fatty acids from chloroplasts [57] and its ortholog is expressed in the mesocarp tissue of avocado (42 RPKM; Additional file 1: Table S2). Thus it is possible that the avocado FAX1 ortholog contributes to export of free fatty acids and that acyl activation may then occur in ER:envelope contact sites or hemifusion [58], consistent with possible ‘channeling’ of acyl groups into phosphatidylcholine (PC) by a lyso-PC acyltransferase (LPCAT; AT1G12640) [59–61]. In this regard, an ortholog of LPCAT, represented by an average of 30 RPKM/stage, was identified in avocado mesocarp (Additional file 1: Table S3). LACS are also responsible for re-esterification of acyl groups generated by phospholipase A2-mediated acyl editing in the ER. To this extent, transcripts for orthologs of three PLA2 isoforms (AT4G29070, AT3G18860, AT2G19690) were detected in avocado mesocarp, which together were represented by an average of 127 RPKM/stage (Additional file 1: Table S3).

Among the other LACS isoforms, *At*LACS2 and *At*LACS3 were shown to be associated with surface lipid synthesis and *At*LACS5 to be floral-tissue specific [52]. While LACS3 was not detectable in avocado mesocarp, both LACS2 and LACS5 orthologs were poorly expressed and therefore less likely to play a role in TAG biosynthesis (Fig. 4). Barely detectable transcripts (<1 RPKM/stage) for orthologs of peroxisomal LACS6 and LACS7 [62, 63] suggest that fatty acids undergo little β-oxidation during mesocarp development in avocado (Fig. 4; Additional file 1: Table S3).

### Most TAG biosynthesis genes in the ER show similar expression patterns among diverse oil-rich tissues

**Fig. 5:**
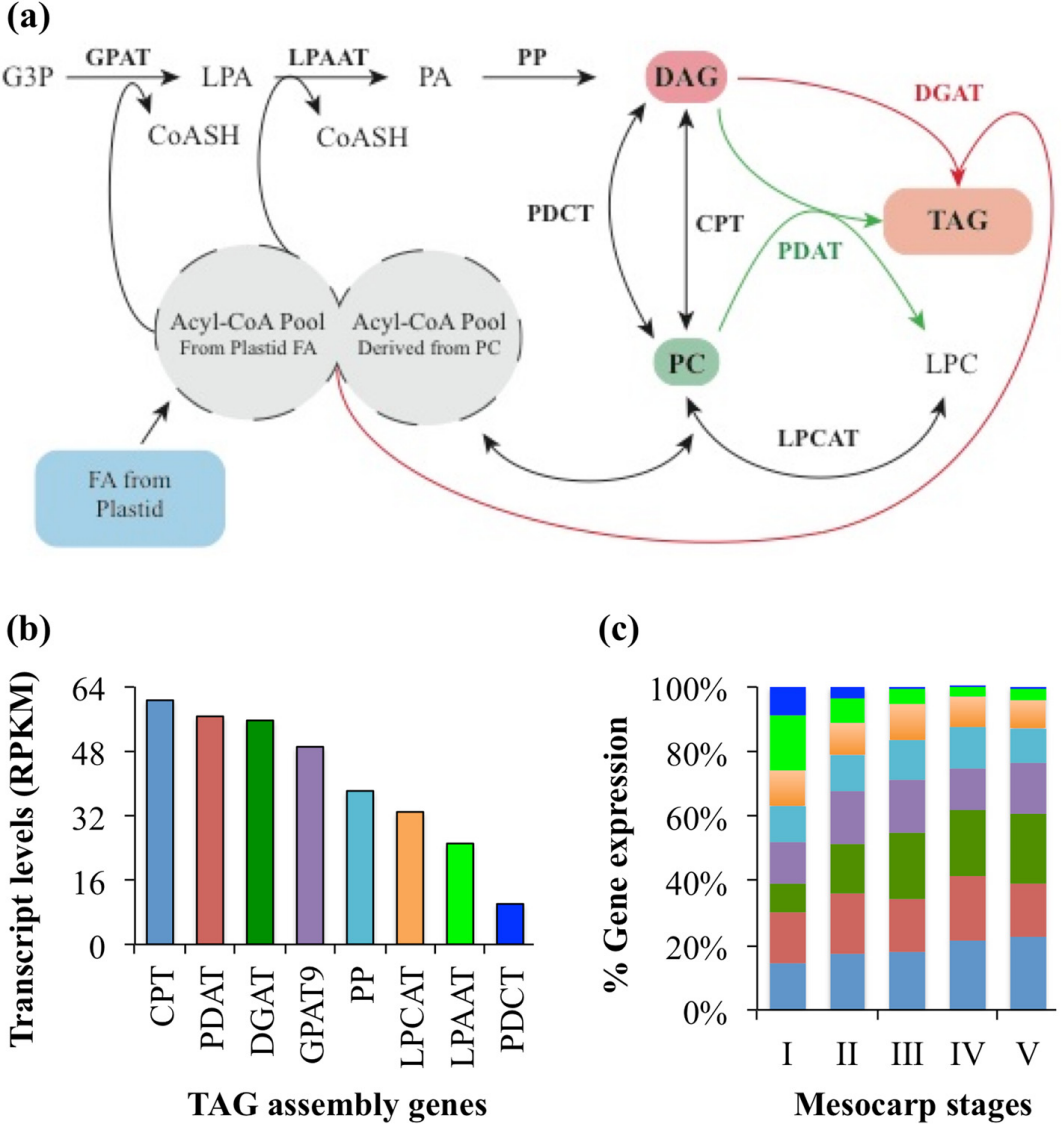
Expression levels for genes associated with triacylglycerol (TAG) assembly. **a** Schematic of TAG pathway. **b** The average transcript levels for each enzyme. **c** The relative distribution of transcript levels for each protein during mesocarp development. The data are expressed as reads per kilobase per million mapped reads (RPKM). The RPKM values for subunits of a protein and for multiple isoforms were summed. Protein abbreviations are provided in Additional file 1: Table S3

In avocado mesocarp, the orthologs of TAG biosynthesis genes (Fig. 5a) were represented by an average of 75 RPKM/protein, which is seven-fold less than that of fatty acid synthesis gene expression per protein (Fig. 2b and 2c). Similar low expression levels for TAG synthesis genes, relative to fatty acid synthesis genes, were noted in other oil-rich seed and nonseed tissues [14, 16]. In avocado mesocarp, the orthologs of all the genes involved in sequential acylation of glycerol-3-phosphate (G3P), *via* the Kennedy pathway to generate TAG, i.e. the glycerol-3-phosphate acyltransferase (GPAT9), lysophosphatidic acid acyltransferase (LPAAT), phospatidate phosphatase (PP/PAH), and diacylglycerol acyltransferase (DGAT) were expressed during mesocarp development (Fig. 5; Additional file 1: Table S3). The expression levels for the ortholog of GPAT9 remained similar during mesocarp development, but declined by two-fold for LPAAT2, the predominant LPAAT isoform in the ER (Additional file 1: Table S3; Fig. 5). Among the various orthologs that encode for PP/PAH, transcripts for PAH1 and PAH2 were abundant and their levels remained moderately similar during the development of mesocarp (Additional file 1: Table S3).

Based on the source of the acyl groups that are available for the acylation of diacylglycerol (DAG) in the terminal step to TAG synthesis, the reactions were referred to as acyl-CoA-dependent or -independent (Fig. 5). The key step in acyl-CoA-dependent TAG synthesis is catalyzed by DGAT. Between the two predominant DGAT forms, DGAT1 was most highly expressed in avocado mesocarp with more than two-fold increase from stages I to V (Fig. 5c). Transcripts for DGAT2 were also detectable but were eight-fold less abundant than those of DGAT1 (Additional file 1: Table S3). In oilseeds, although the expression of genes involved in TAG synthesis remained relatively low, the expression levels for DGAT were an exception. In rapeseed and castor, relative to GPAT9, the DGAT isoforms were expressed seven- and nine-fold higher, respectively, and the increase in DGAT transcript levels coincided with their oil accumulation (Additional file 1: Table S4; [14]. In contrast, although both DGAT1 and DGAT2 were abundantly expressed in the mesocarp of oil palm, which accumulates about 80–90 % TAG, the transcript levels, on average, were only two-fold higher than that of GPAT9 (Additional file 1: Table S4; [16]. Similarly, in avocado, the DGAT transcript levels were comparable to that of GPAT9 (Additional file 1: Table S4).

### Flux through PC might play an additional role in TAG accumulation in avocado mesocarp

Multiple pathways exist in plants for the assembly of TAG in the ER and it has been particularly challenging to decipher the relative flux through the alternatives [13, 64]. In addition to *de novo* DAG that is generated *via* the Kennedy pathway, DAG precursors for TAG synthesis can also be derived from PC by the reversible action of two enzymes, PC:DAG cholinephosphotransferase (PDCT/ROD1; [65] and/or cytidine-5′-diphosphocholine:DAG cholinephosphotransferase (CPT; [66, 67]. In avocado mesocarp, the expression levels for the ortholog of *At*CPT were on average six-fold higher than that of PDCT (Fig. 5b; Additional file 1: Table S3). In addition, in avocado and also in oil palm, but in contrast to oilseeds, an ortholog of phospholipid diacylglycerol acyltransferase (PDAT1; AT5G13640) showed transcript levels that were comparable to that of DGAT (Fig. 5; Additional file 1: Table S4). Furthermore, for rapeseed and castor seed tissues, where DGAT levels were predominant, the PDAT1 transcripts were expressed at low levels relative to GPAT9 (Additional file 1: Table S4). Previously, Stobart and Stymne concluded that TAGs are synthesized predominantly *via* the Kennedy pathway in avocado since their microsomes were deficient in acyl exchange and interconversion of DAG to PC [68]. While it is possible that DAG:PC exchange and PDAT do not contribute to a major flux in oleaginous mesocarp of avocado, particularly in postharvest ripening stage [68], the transcript levels for CPT and PDAT, relative to other oil-rich tissues (Additional file 1: Table S4) suggest the possibility for PC as an intermediate in avocado TAG synthesis, particularly during early fruit development and needs to be further investigated.

Typically, acyl flux into PC is rapid by ‘acyl exchange/editing’ processes, which allow for further modification, such as desaturation. In avocado mesocarp, while about 18 % of the total lipids are polyunsaturated in stages I to III, less than 10 % are polyunsaturated in stages IV and V (Fig. 1c). Coinciding with the lipid composition, the higher transcript levels for LPCAT and PDAT in stages I to III, relative to IV and V (Additional file 1: Table S3) suggest a possible role for acyl editing in the early stages of mesocarp development. Consistent with this, the transcript levels for an ortholog of oleate desaturase (FAD2) were also more than two-fold higher in the earlier stages of development, relative to stages IV and V (Additional file 1: Table S3). The FAD2 transcript levels were however, on average only 1.5 times higher than that of GPAT9 (Additional file 1: Table S4), reflecting the overall oleaginous nature of avocado mesocarp. In contrast, the FAD2 transcript levels in rapeseed, castor and oil palm were 46, 49 and 144 times higher, respectively, relative to GPAT9 (Additional file 1: Table S4). Collectively, these results suggest that in avocado mesocarp and other nonseed tissues, flux through PC may play an additional role in achieving high amounts of TAG accumulation.

### Proteins different from that of seed tissues likely coat lipid droplets in avocado mesocarp

Lipid droplet proteins such as oleosins, caleosins, steroleosins have been widely recognized for their role in compartmentalization of storage lipids, both in seed and some nonseed tissues, such as anther and pollen [69–72]. Recently, proteomics, lipidomics and transcriptomics contributed to the elucidation of two new lipid droplet-associated proteins (LDAP1 and LDAP2, homolog of At3g05500) in avocado mesocarp [20, 21]. The summed transcript levels for LDAP1 and LDAP2 were more than 250 RPKM, on average, across the five developmental stages of the mesocarp (Additional file 1: Table S2; [20]. These proteins have homology to small rubber particle proteins and are predicted to bind to and stabilize lipid-rich particles in avocado mesocarp tissues. The lipid droplets of avocado and other oil rich tissues are much larger than in oilseeds; in mesocarp of oil palm the lipid droplets fuse when the tissue is homogenized [16]. Previous transcriptome studies showed that oil-rich mesocarp tissues of oil palm and olive barely expressed transcripts for oleosins, caleosins and steroleosins and were considered unlikely to play a significant role in stabilization of TAG during fruit development [15, 16, 18]. Similarly, in avocado mesocarp, although some of the orthologs for oleosins (At3G18570), caleosins (At1G70670; At2G33380), and steroleosins (At5G50700) were detectable their transcript levels were very low (<10 RPKM; Additional file 1: Table S3), supporting a conclusion that these seed-associated proteins are unlikely to participate in stabilizing lipids in nonseed tissues.

### Multiple orthologs of WRI are highly expressed in avocado mesocarp

Transcriptome studies of oil palm mesocarp revealed that *WRI1*, in addition to its high expression in seeds, is also highly expressed in correlation with oil accumulation in nonseed tissue [16, 18, 32, 33]. Interestingly, in avocado mesocarp, in addition to *WRI1*, transcripts for its isoforms WRI2 and WRI3 were also highly expressed. Furthermore, as in oil palm mesocarp, the orthologs of upstream regulators of *WRI1* in seed tissues, such as LEC1, LEC2, and FUS3 were either not expressed or barely detectable in avocado mesocarp (Additional file 1: Table S3). Transcripts for ortholog of ABI3 (At3g24650) were, however, on average 43 RPKM (Additional file 1: Table S3). These data reinforce the conclusion that WRI1 in nonseed tissues is likely regulated differently than in seed tissues.

**Fig. 6:**
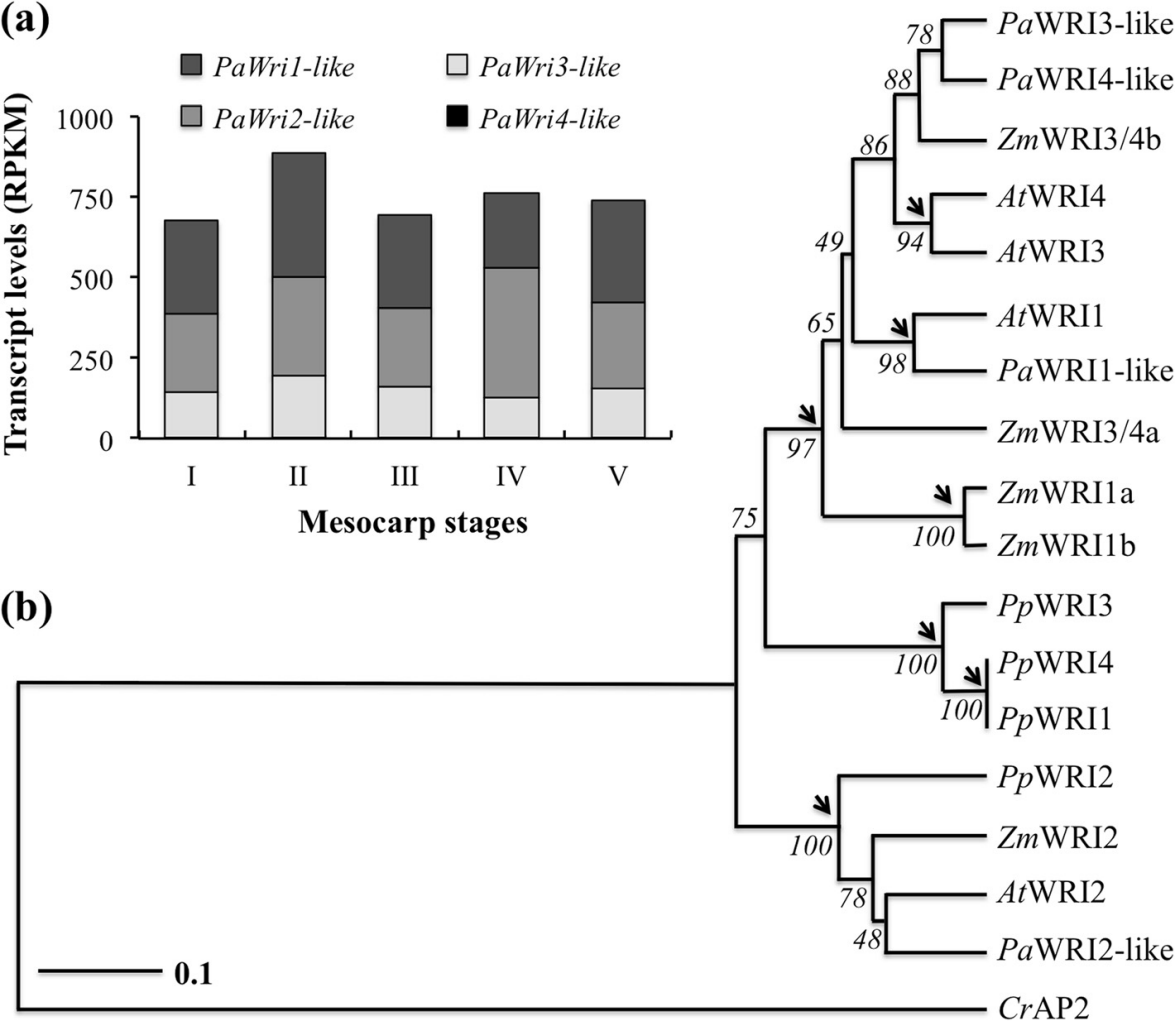
Expression and phylogenetic analysis of Wrinkled (WRI) isoforms (**a**) Transcript levels for *PaWRI-like* isoforms in developing mesocarp of avocado. **b** Phylogenetic analysis of *At*WRI orthologs in *Oryza sativa*, *Physcomitrella patens* and *Persea americana*. An AP2 transcription factor from *Chlamydomonas reinhardi* was used as outgroup. Bootstrap values for 1,000 replicates are indicated and arrows point to possible duplication events

Recent studies in *Arabidopsis* showed that *WRI3* and *WRI4* can each compensate for the low fatty acid levels of the *wri1-4* mutant; they are non-redundant in function and are required in floral tissues for cutin biosynthesis [31]. Interestingly, in avocado mesocarp, the overall expression pattern of *WRI* orthologs was similar to that of genes that WRI1 is known to regulate such as *ACP*, *BCCP*, *KASII*, and *PDHC* (Fig. 3a; [30] and to the pattern of oil accumulation (Figs. 1b and 6a). Although complementation and transcriptional activator studies ruled out the role of WRI2 in *Arabidopsis* fatty acid biosynthesis [31], the high expression levels of its ortholog in mesocarp tissue of a basal angiosperm, during oil accumulation, suggest that it may have a role in nonseed tissue. A phylogenetic tree generated from WRI homologs, from various plant families including dicots, monocots, and a basal angiosperm, revealed a possible gene duplication event of WRI early in land plant evolution as the WRI2 proteins formed a monophyletic group and separated from all other WRI homologs. Other WRI homologs formed two distinct groups with a clade of WRI genes all belonging to *P. patens*, a bryophyte, separated from the WRI1, WRI3 and WRI4 genes of higher plants (Fig. 6b). The tree constructed for the WRI genes of various species suggests that the *PaWRI2-like* and *AtWRI2* are older than the other WRI genes and have also diverged a great deal from each other. The high expression levels for *WRI2-like* in avocado mesocarp that were not previously reported in any other oil-rich tissues, along with WRI1 and WRI3 but not WRI4, suggest that perhaps through divergence, the *At*WRI2 may have departed its function in oil biosynthesis while the *PaWRI2-like* retained its function. Although *AtWRI2* did not complement *wri1* mutant, complementation studies with *PaWRI2-like* are underway. Based on the gene expression data, it is predicted that *WRI2* homolog of avocado may play an additional role in TAG accumulation in this basal angiosperm species.

### The available carbon for oil biosynthesis in avocado fruit may be atypical

**Fig. 7:**
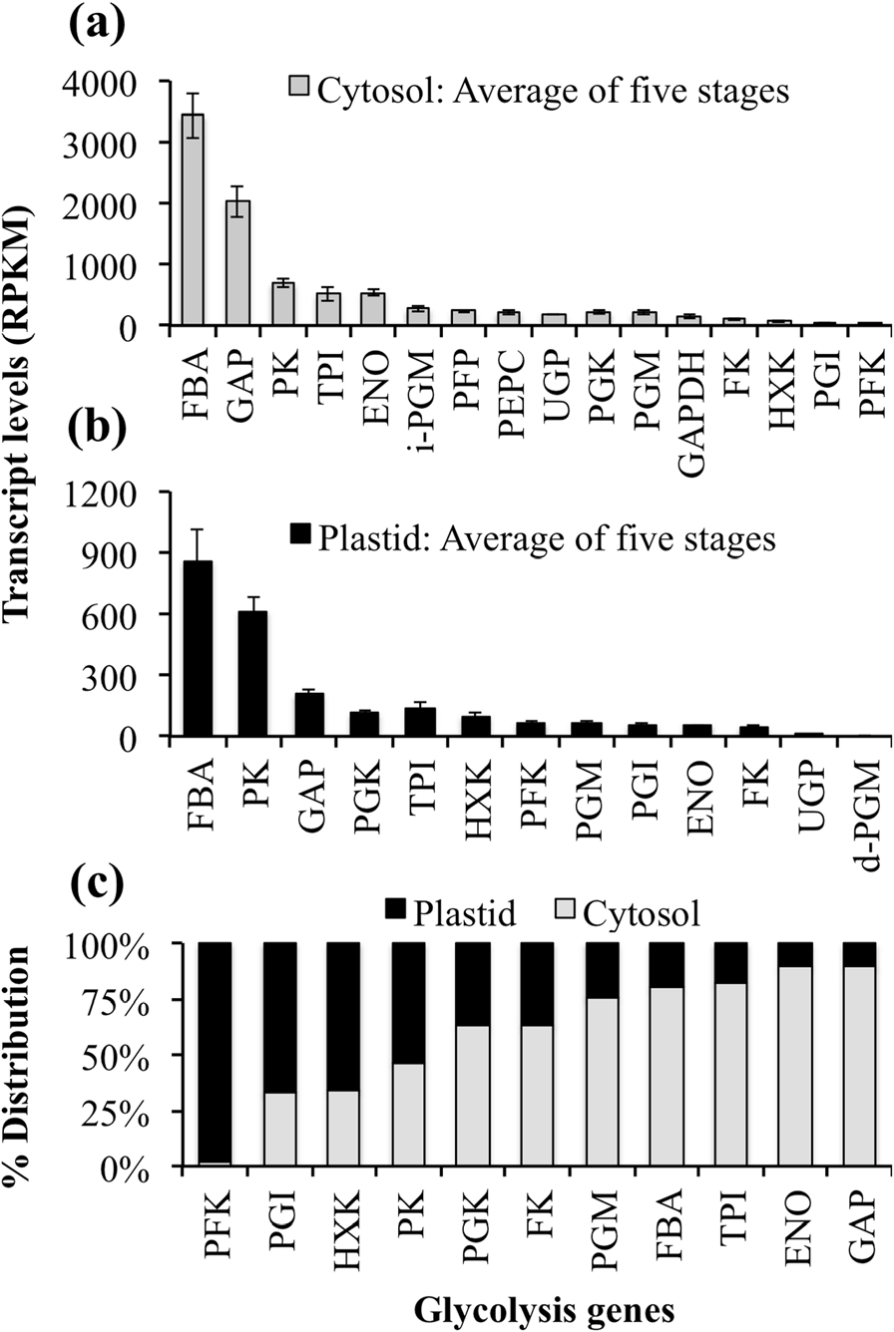
Transcript levels for genes associated with glycolysis. **a** The expression levels for cytosolic glycolysis genes. **b** The expression levels for plastidial glycolysis genes. **c** The relative distribution of glycolysis genes in plastid and cytosol. The data, reads per kilobase per million mapped reads (RPKM), are average transcript levels of five developing stages of mesocarp with error bars representing their standard deviation. The RPKM values for subunits of a protein and for multiple isoforms were summed. Protein abbreviations are provided in Additional file 1: Table S3

Several studies showed that an increase in oil content in mesocarp during fruit growth is accompanied by a decrease in the concentration of reducing sugars [73–75]. Typically, photosynthates are transported into the mesocarp in the form of sucrose *via* the sugar transporters between the apoplast, cytosol and vacuole and are hydrolyzed to hexoses that are utilized as the carbon source for glycolysis. The transcripts that likely encode for sugar transporters in the plasma membrane and vacuolar membranes were however, poorly expressed in avocado mesocarp with an average of 26 RPKM across all the developmental stages (Additional file 1: Table S3). The avocado fruit, especially in its early stages of growth, in addition to fixation of CO_2_*via* ribulose 1,5 bisphosphate carboxylase (RBC), is also capable of carboxylation of phosphoenolpyruvate (PEP) with bicarbonate that is available in the intercellular space of the fruit by PEP carboxylase (PEPC) to produce oxaloacetate and subsequently to malate [76–79]. Akin to mechanisms found in the leaves of C4 and CAM plants, malic enzyme can recycle CO_2_ produced in the non-green mesocarp layers for subsequent fixation by RBC in the more green tissues and concurrently release pyruvate for fatty acid synthesis. Consistent with this notion, the transcript levels for the orthologs of RBC, phosphoribulokinase and PEPC were high and on average 183, 195 and 216 RPKM, respectively (Additional file 1: Table S3). The abundance of these transcripts, particularly for the ortholog of PEPC in the mesocarp tissue during its development is consistent with its suggested role in carbon assimilation (Fig. 7a).

The transcripts for the enzymes that hydrolyze sugars, such as sucrose synthase (SuSy) in the cytosol were highly expressed with more than 500 RPKM/protein, on average, during mesocarp development (Additional file 5: Figure S3a; Additional file 1: Table S3), implicating that SuSy might be the major player in generation of hexoses necessary for pyruvate synthesis. Among the invertases, however, the transcript levels for vacuolar invertases were also abundant (Additional file 5: Figure S3a, Additional file 1: Table S3). Typically, acid invertases hydrolyze the sucrose stored in vacuoles and the hexoses generated might be transported to the cytosol by a transporter or *via* facilitated diffusion [80]. It remains to be determined if in avocado mesocarp the hexoses from vacuoles might also undergo glycolysis. Starch is also a principal substrate for glycolysis in the plastids and in avocado mesocarp, transcripts for starch synthesis and degradation gene orthologs were abundant throughout mesocarp development (Additional file 5: Figure S3c). In the early stages of fruit set (June), about 44 % of the flesh weight is contributed by the sugars, which continue to increase during the rapid growth period of the fruit (until late October) and then begin to decline during the period of oil accumulation [81]. At maturity, the total carbohydrates in mesocarp contribute to about 10 % of the flesh weight and are composed of about 10 % starch, 20 % sucrose, 10 % hexoses and 60 % C7 sugars and sugar alcohols (mannoheptulose and perseitol; [8]. Sedoheputlose-7-P is produced by the activity of transketolase (TK) and is further converted to mannoheptulose by transaldolase (TA); it is not clear if mannoheptulose is exclusively derived from translocated sugars or is also synthesized in the mesocarp. Both TK and TA orthologs showed higher expression levels in mesocarp plastids, with TA being two-fold higher than TK (Additional file 5: Figure S3c), suggesting a possibility for their synthesis in mesocarp as well. The higher levels of C7 sugars in early stages of fruit growth might therefore play a role in regulating the initiation of oil biosynthesis. Their presence at maturity was considered necessary as respiratory metabolites for post-harvest fruit ripening [8, 82].

### Plastidial and cytosolic glycolysis may cooperatively generate the pyruvate necessary for fatty acid synthesis

The degradation of sucrose followed by glycolysis and transport of its intermediates to the plastids is crucial for providing carbon for fatty acid synthesis (Fig. 2a). Transcriptome analysis of avocado mesocarp indicates that a complete glycolytic pathway likely occurs in both cytosol and plastids (Fig. 7; Additional file 1: Table S3). Additionally, the high expression levels for several orthologs that likely encode for plastid transporters also indicate that the intermediates, hexoses and triose carbons or PEP, and pyruvate generated by the cytosolic glycolytic pathway may be transported to the plastid (Additional file 5: Figure S3b) [83, 84]. Decarboxylation of imported malate by a plastidial NADP-dependent malic enzyme (NADP-ME) is also an alternate route for generation of pyruvate, as reported in castor endosperm [85] and maize [86]. Although the expression levels for ortholog of cytosolic malic acid dehydrogenase (MDH) were fairly abundant (>60 RPKM, Additional file 1: Table S3), transcripts for NADP-ME were poorly represented in the plastid (<10 RPKM, Additional file 1: Table S3). These data suggest that malate synthesis in cytosol and its import to plastid for further decarboxylation might not generate substantial pyruvate in the plastids of avocado mesocarp (Additional file 1: Table S3).

Comparing the transcript levels for the orthologs of glycolytic enzymes in the plastid and cytosol revealed features that support the generation of pyruvate in the plastid necessary to drive fatty acid synthesis during mesocarp development (Fig. 7). In both cytosol and plastid, the orthologs for glycolysis enzymes were highly represented (>600 RPKM/enzyme; Fig. 2c), with putative fructose-bisphosphate aldolase (FBA) being the most abundantly expressed gene (Fig. 7a and b). Glycolysis is, however, primarily regulated by those enzymes that catalyze the reactions involved in the conversion of hexose to hexose-P, fructose-6-P to fructose-1,6-diP, and PEP to pyruvate [87]. The abundance of transcript levels for orthologs of UDP-glucose pyrophosphorylase (UGPase), fructokinase, and pyrophosphate-dependent phosphofructokinase in the cytosol (Additional file 6: Figure S4 and Fig. 7), along with high transcript levels for SuSY and invertases (Additional file 5: Figure S3a) suggest that cytosolic glycolysis is highly active and might rely more on UGPase generated fructose as a substrate. Interestingly, the higher abundance of transcript levels for the orthologs of hexokinase, glucose-6-phosphate isomerase, which catalyzes the conversion of glucose-6-P to fructose-6-P and ATP-dependent 6-phosphofructokinase in the plastid than in the cytosol (Additional file 6: Figure S4 and Fig. 7c), suggests the early glycolysis is highly active in plastid as well and perhaps relies primarily on glucose as the substrate. Furthermore, the abundant gene expression levels for the orthologs of plastidial transporters for glucose (GLT), glucose-6-P (GPT), and nucleotide (NTT) through out the mesocarp development (>100 RPKM; Additional file 5: Figure S3) suggests the scope for transport of glycolysis precursors and intermediates to the plastid. The high expression levels for pyruvate kinase in the plastid (Fig. 7) additionally suggests that late glycolysis in oil-rich tissues of avocado might be under plastidial control. Overall, the means to generate pyruvate for fatty acid synthesis in plastid in a basal angiosperm species appears to be a synergistic outcome of active glycolysis in both the cytosol and plastid and transport of intermediates to the plastid, similar to those observations made with oil-rich dicot and monocot tissues [14, 16].

## Conclusions

Avocado, as a basal angiosperm with highly nutritious fruit that is rich in oleic acid in its nonseed tissue, serves as an elegant system for comparing TAG biosynthesis functions among oil-rich tissues of diverse angiosperms. In this study, avocado mesocarp gene expression was examined with a focus on pathways and regulators responsible for the supply of carbon and its conversion to oil in nonseed tissue. We also addressed overall evolutionary conservation of genes required for oil synthesis across multiple oil-rich species. In general, genes expressed in processes from sucrose degradation to TAG assembly that are known to be upregulated in oil-rich tissues of monocots and dicots [14, 16], were also upregulated in avocado mesocarp (Fig. 2). Furthermore, consistent with other studies for oil-rich tissues, the expression of transcripts for fatty acid biosynthesis was several fold higher than those of transcripts encoding later steps of TAG assembly in the ER (Figs. 2, 3, and 5). Plastid genes and transporters, necessary for pyruvate generation, were also highly expressed in the mesocarp tissue (Fig. 7). Most notably, transcripts for orthologs of multiple WRI isoforms were also abundant in the oil-rich tissues of avocado (Fig. 6). Together, these data indicate that the supply of carbon and perhaps regulation of oil biosynthesis may primarily occur in the plastid in basal angiosperms as well. Further complementation studies are essential to establish the function of various isoforms of WRI in nonseed tissues. Comparative analysis of transcription factors, expressed across various oil-rich tissue types and species, is necessary to identify potential candidates that may play the role of upstream regulators to WRI.

Quantitative analysis of avocado mesocarp transcriptome also revealed certain unique features that suggest further studies using avocado to address several gaps in our understanding of TAG synthesis in nonseed tissue, such as regulation and determination of oil composition. For example, it is noteworthy that within the ER, the most abundant transcripts, relative to GPAT9 in avocado mesocarp, were of LACS orthologs (Fig. 4, Additional file 1: Table S3 and Additional file 1: Table S4) suggesting the potential for acyl activation in the ER and/or the junction of ER and plastid. Oil-rich nonseed tissues of avocado may therefore offer an invaluable system to determine roles for plastid versus ER associated LACS activity and/or if a direct contact between the plastid and ER [58] exists in basal angiosperms. Furthermore, avocado mesocarp could be used to determine the preference for PDAT1 and to explore its overlapping function with DGAT1 in TAG synthesis. This oleaginous species also is suitable to address if acyl editing occurs in mesocarp, where there is little flux to desaturation, and if it either involves phospholipase 2 and LACS or is mediated by LPCAT. With the absence or poor expression of oil storage protein such as oleosins, if or how TAG is packaged in nonseed tissues has remained a mystery; the identification of LDAP1 and LDAP2 in avocado mesocarp, however, offers an alternative means to study the stabilization of TAG.

Avocado fruit is distinctive among angiosperms in its development and growth, particularly in aspects that include the nature of storage metabolites it accumulates. The role of 7-carbon sugars and starch, in the early stages of mesocarp development, in regulation of fruit ripening and possibly in initiation of lipid synthesis remains elusive. Comprehensive profiling of carbohydrate, lipid and hormone content, concurrent with transcriptomics of mesocarp and seed tissues, is expected to provide a more in-depth understanding of the coordinated process of fruit development and carbon partitioning.

## Methods

### Plant material

Avocado fruits (cv. Hass) were harvested from a tree (44-15-11 Hass Scion on D7 clonal rootstock) during October 2009 to February 2010 and were shipped overnight at 4 °C to Michigan State University. The clonal stocks are located at University of California South Coast Research and Extension Center in Irvine, CA. Fruits from five stages were weighed and dissected to separate epicarp, mesocarp and seed (Additional file 1: Table S1; Fig. 1). The isolated tissues were weighed and flash frozen in liquid N_2_ and stored at −80 °C until further use.

### Lipid extraction and quantification

To determine the fatty acid content and composition of avocado fruit tissues (mesocarp and seed), their total lipids were extracted with hexane-isopropanol method [88]. Extracted lipids were weighed and resuspended in hexane and converted to fatty acid methyl esters, by a base-catalyzed methylation reaction [89], and analyzed using gas chromatography coupled with flame ionization detector (Varian 3800), to determine the fatty acid composition [90]. Fatty acids were quantified against triheptadecanoin that was added as an internal standard prior to lipid extraction.

### Total RNA extraction, cDNA library construction and sequencing

Total RNA was extracted from 3 g of mesocarp tissue that had been ground finely in liquid N_2_ and incubated for 10 min in 30 mL of TRIzol® reagent (Life technologies) and for an additional 5 min with 6 ml of CHCl_3_. After centrifugation at 12,000 *g* for 15 min at 4 °C, the aqueous phase was incubated overnight with 1/3 volume of 8 M LiCl. Samples were then centrifuged at 12,000 g for 30 min at 4 °C and the pellet was resuspended in 1000 μL of RLT buffer of RNEasy kit (Qiagen) and RNA was eluted following the manufacturer’s protocol.

RNA-seq data for developing mesocarp were generated using Illumina sequencing techniques. Two technical replicates (a and b) for stage I and stage III were included for RNA-seq (Additional file 1: Table S1). RNA quality was assed using the Agilent BioAnalyzer (Agilent Technologies) and all samples submitted for sequencing had a RIN score of 6.4 or higher. Libraries were created using an Illumina pre-release protocol for directional mRNA-seq library prep (v1.0). A single read 75 cycle run was then performed on the Illumina GAIIx sequencer, following manufacturers protocols. Reads were trimmed and filtered based on quality with the Trim Sequences algorithm of CLC Genomics Workbench software (Limit: 0.05, Maximum ambiguities: 2). Details on the RNA-seq datasets (Additional file 1: Table S1) are available in the NCBI Short Read Archive within BioProject PRJNA253536 (http://www.ncbi.nlm.nih.gov/bioproject/253536).

For 454 sequencing, mRNA was isolated from the total RNA using Sera-Mag Oligo (dT) Magnetic Beads (Thermo Scientific). cDNA libraries were generated from pooled samples (five stages plus two technical replicates) using the Roche cDNA Rapid Library Prep Kit (Roche Diagnostics). Sequences were obtained on the Roche 454 GS FLX sequencer using the titanium chemistry (Roche Diagnostics).

### Bioinformatics and data analyses

A reference designed for comparative mapping of the mesocarp RNAseq reads was prepared using Trinity v.2 [91] for *de novo* assembly with inputs of the above Illumina reads plus 454 and Illumina paired reads generated from sequencing of Hass leaf and flower mRNA of an independent project, whose data and details are provided under NCBI BioProject PRJNA258225. This allowed for more complete transcript references than using the mesocarp single read Illumina data alone. This generated 151,788 contigs that were then clustered using CD-HIT-EST with default parameters (sequence identity: 90 %, word size: 10), resulting in 134,329 sequence clusters (Additional file 1: Table S1). Sample expression was estimated using CLC Genomics Workbench version 5.5.1. Unique counts were generated by aligning the RNAseq reads to the assembled contigs using the RNA-Seq Analysis algorithm for non-annotated sequences (Parameters: Similarity 0.8; Length fraction 0.75).

The RPKM values obtained by Illumina sequencing were highly correlated between the technical replicates of stage 1a and 1b (R^2^ = 0.96702; Additional file 7: Figure S5a) and stage 3a and 3b (R^2^ = 0.97526; Additional file 7: Figure S5b). About 250 gene orthologs that are likely associated with lipid metabolism were considered in this study and their transcript levels obtained by 454 sequencing, where all the samples were pooled, were also highly correlated with average expression data for all the five mesocarp stages obtained by Illumina sequencing (R^2^ = 0.91171; Additional file 7: Figure S5c).

### Phylogenetic analyses

Evolutionary relationship of *WRI* genes in a monocot (maize), dicot (arabidopsis), basal angiosperm (avocado) and bryophyte (*Physcomitrella patens*) was analyzed by construction of a phylogenetic tree. The protein sequences for four AtWRI genes were identified from the TAIR database and the avocado homologs were obtained from the transcriptome data (Additional file 1: Table S1). A UPGMA tree was constructed with MEGA 6.0 using a ClustalW alignment of protein sequences [92]. The robustness of the tree was tested by bootstrap analysis with 1,000 replicates. The orthologs of AtWRI1 in maize and moss were identified using BLASTP (NCBI). In maize, two sequences that were homologous to AtWRI3 and AtWRI4 were almost identical and were referred to as WRI3/4. Also maize is known to have a species-specific duplication of the WRI1 gene and both function to regulate fatty acid synthesis [93]. An AP2 transcription factor from *Chlamydomonas reinhardi* was used as an outgroup for the WRI tree.

### Accession numbers

AtWRI1 (NP_001030857.1); AtWRI2 (NP_001189729.1); AtWRI3 (NP_563990.1); AtWRI4 (NP_178088.2); ZmWRI1a (NP001137064.1); ZmWRI1b (NP_001131733.1); ZmWRI2 (NP_001145827.1); ZmWRI3/4a (XP_008656570.1); ZmWRI3/4b (XP_008651355.1) (PpWRI1-like (BAL04570.1); PpWRI2-like (XP_001765028.1); PpWRI3-like (XP_001770958.1); PpWRI4-like (XP_001764166.1); CrAP2 (XP_001699213.1).

### Availability of supporting data

The supporting data associated with this publication are included as additional files. RNA-seq data with details of datasets are available on the NCBI Short Read Archive Project - PRJNA253536 (http://www.ncbi.nlm.nih.gov/bioproject/253536).

## Additional files

Additional file 1: Table S1. Summary of RNA-seq data with details of datasets available on the NCBI Short Read Archive. **Table S2.** Contigs represented by at least 10 reads per kilobase per million mapped reads (RPKM) and their annotation in relation to *Arabidopsis* proteins. **Table S3.** Annotation and transcript levels for select genes associated with conversion of sucrose to triacylglycerol, as shown in Fig. 2. **Table S4.** Transcript levels for genes associated with TAG assembly in the ER, relative to *GPAT9* expression in avocado mesocarp (*Pa* Me), rapeseed embryo (*Bn* Em), castor endosperm (*Rc* En), oil palm mesocarp (*Eg* Me), and date palm mesocarp (*Pd* Me). (XLSX 1350 kb) Additional file 2:
**Data S1.** The sequence information for contigs represented by at least 10 reads per kilobase per million mapped reads (RPKM) and their annotation in relation to *Arabidopsis* proteins. (TXT 13061 kb) Additional file 3: Figure S1. Correlation (R^2^) of lipid content in avocado mesocarp with that of total fruit weight during development. (TIFF 14823 kb) Additional file 4: Figure S2. Transcript levels for plastidial fatty acid synthesis genes, relative to KASIII in avocado mesocarp (*Pa* Me), rapeseed embryo (*Bn* Em), castor endosperm (*Rc* En), oil palm mesocarp (*Eg* Me), and date palm mesocarp (*Pd* Me). (TIFF 14823 kb) Additional file 5: Figure S3. Transcript levels for genes associated with carbon metabolism. (a) sucrose degradation, (b) transport of glycolysis intermediates and (c) starch and mannoheptulose metabolism. The RPKM values for subunits of a protein and for multiple isoforms were summed. Protein abbreviations are provided in Additional file 1: Table S3. (TIFF 14823 kb) Additional file 6: Figure S4. Comparison of transcript levels for genes associated with early glycolysis in cytosol (C) and plastid (P). (a) UDP-glucose pyrophosphorylase (UGPase), (b) hexokinase (HXK), (c) fructokinase (FK) and (d) pyrophosphate-dependent phosphofructokinase (PFP) and ATP-dependent 6-phosphofructokinase (PFK). In plastids, PFK is predominant but PFP is absent. (TIFF 14823 kb) Additional file 7: Figure S5. Correlation analyses of RNA-seq data. (a) Correlation between the technical replicates of stage 1, and (b) and stage 3, obtained by Illumina sequencing. (c) Correlation between the 454 data and Illumina data obtained for ~ 250 gene orthologs used in the current study. (TIFF 14823 kb)
