# Supplementary figures and images for: Oil biosynthesis in a basal angiosperm: transcriptome analysis of Persea Americana mesocarp
Source: BMC Plant Biol. 2015 Aug 16;15:203. doi: 10.1186/s12870-015-0586-2 (PMC4537532; doi:10.1186/s12870-015-0586-2)

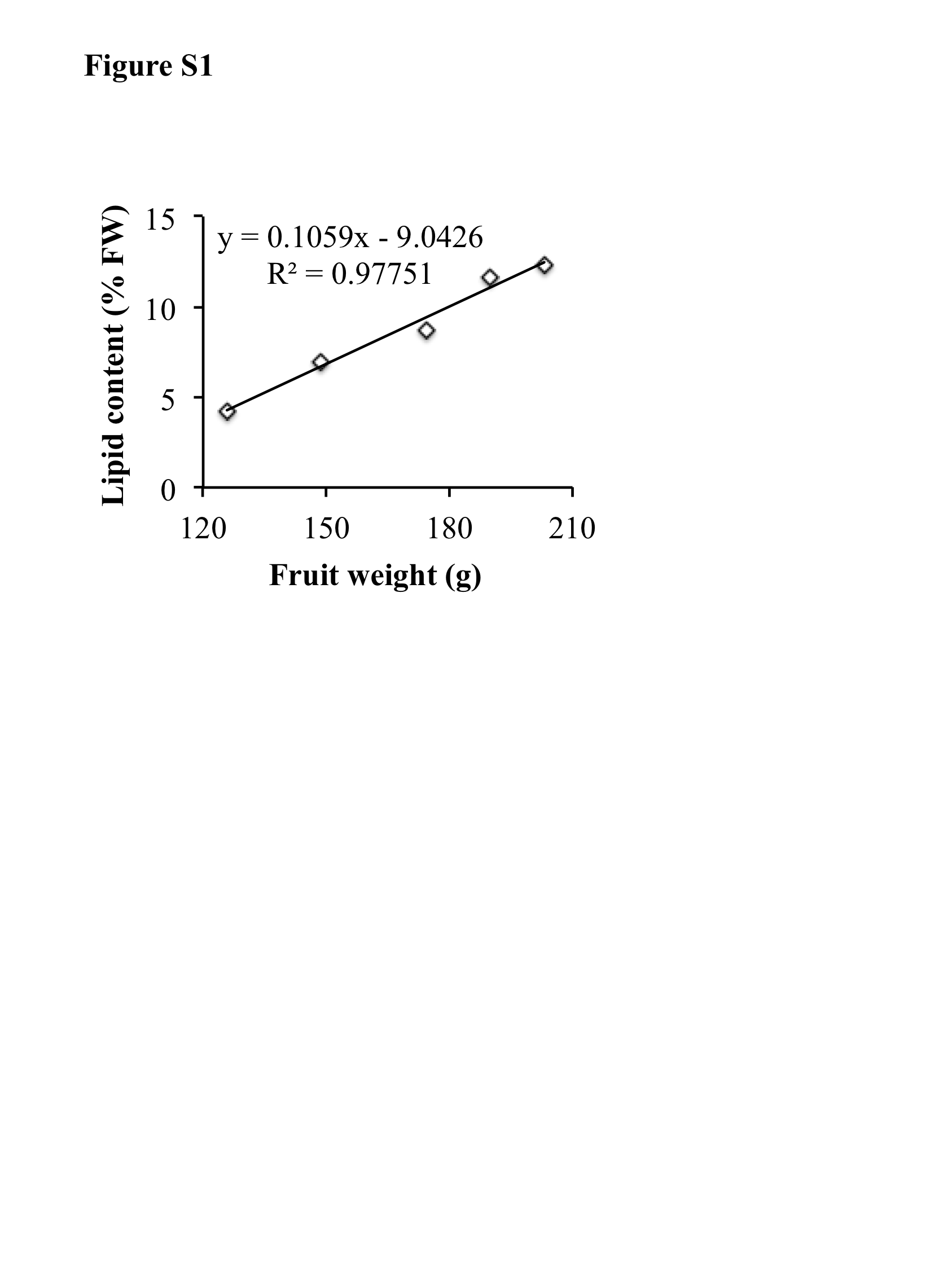

Supplement: Additional file 3: Figure S1. — Correlation (R2) of lipid content in avocado mesocarp with that of total fruit weight during development. (TIFF 14823 kb) [file 12870_2015_586_MOESM3_ESM.tiff]

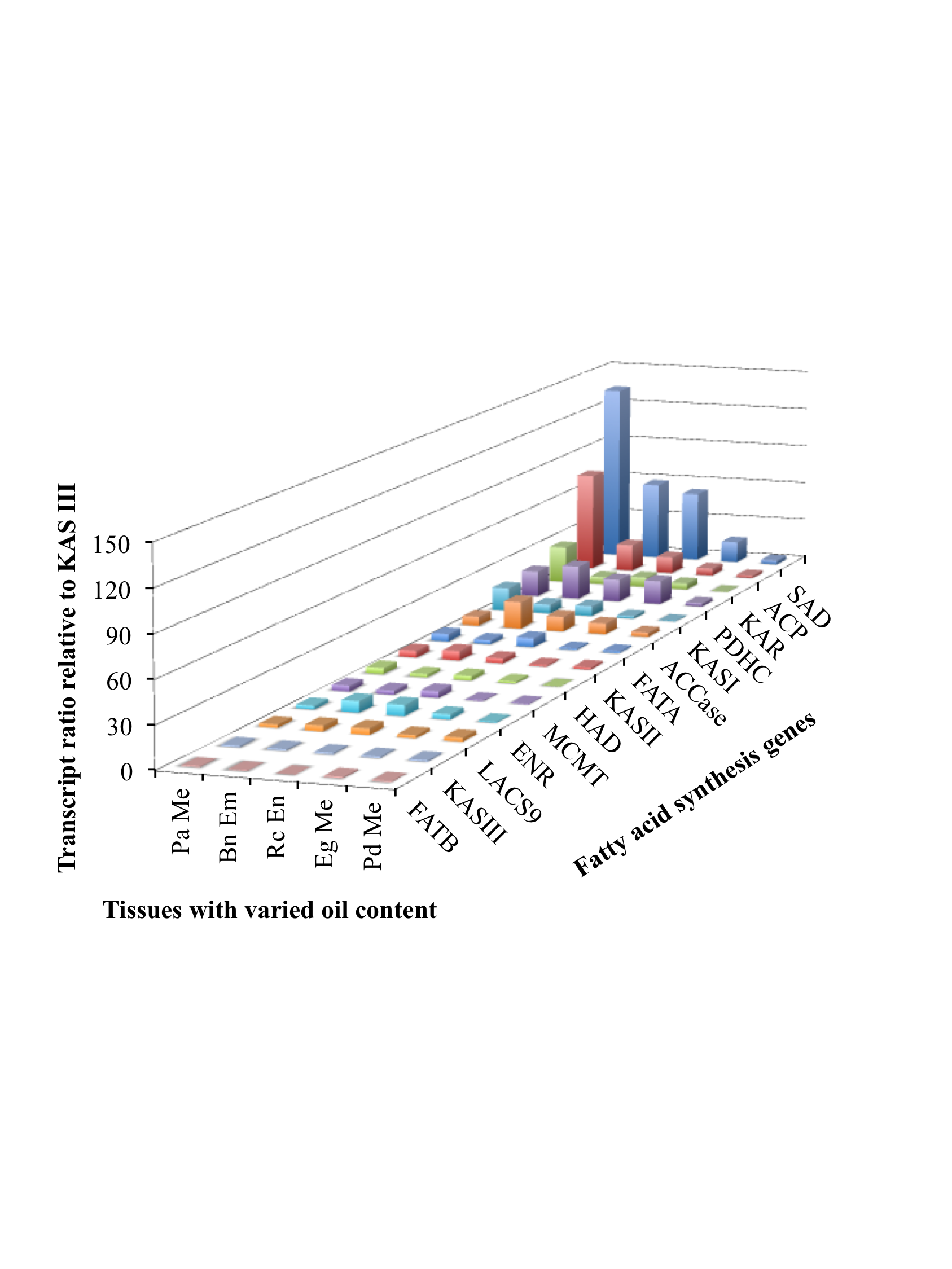

Supplement: Additional file 4: Figure S2. — Transcript levels for plastidial fatty acid synthesis genes, relative to KASIII in avocado mesocarp (Pa Me), rapeseed embryo (Bn Em), castor endosperm (Rc En), oil palm mesocarp (Eg Me), and date palm mesocarp (Pd Me). (TIFF 14823 kb) [file 12870_2015_586_MOESM4_ESM.tiff]

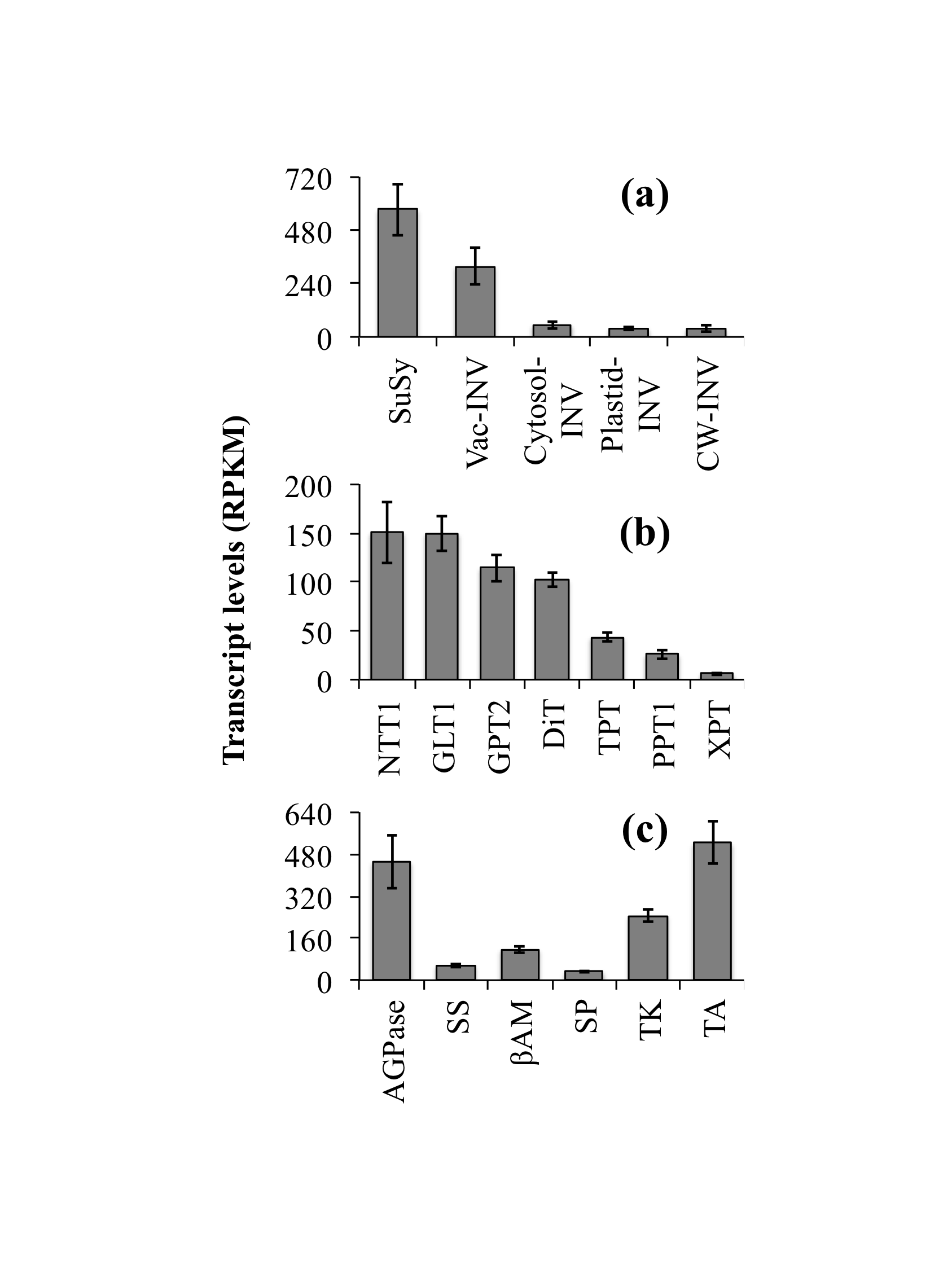

Supplement: Additional file 5: Figure S3. — Transcript levels for genes associated with carbon metabolism. (a) sucrose degradation, (b) transport of glycolysis intermediates and (c) starch and mannoheptulose metabolism. The RPKM values for subunits of a protein and for multiple isoforms were summed. Protein abbreviations are provided in Additional file 1: Table S3. (TIFF 14823 kb) [file 12870_2015_586_MOESM5_ESM.tiff]

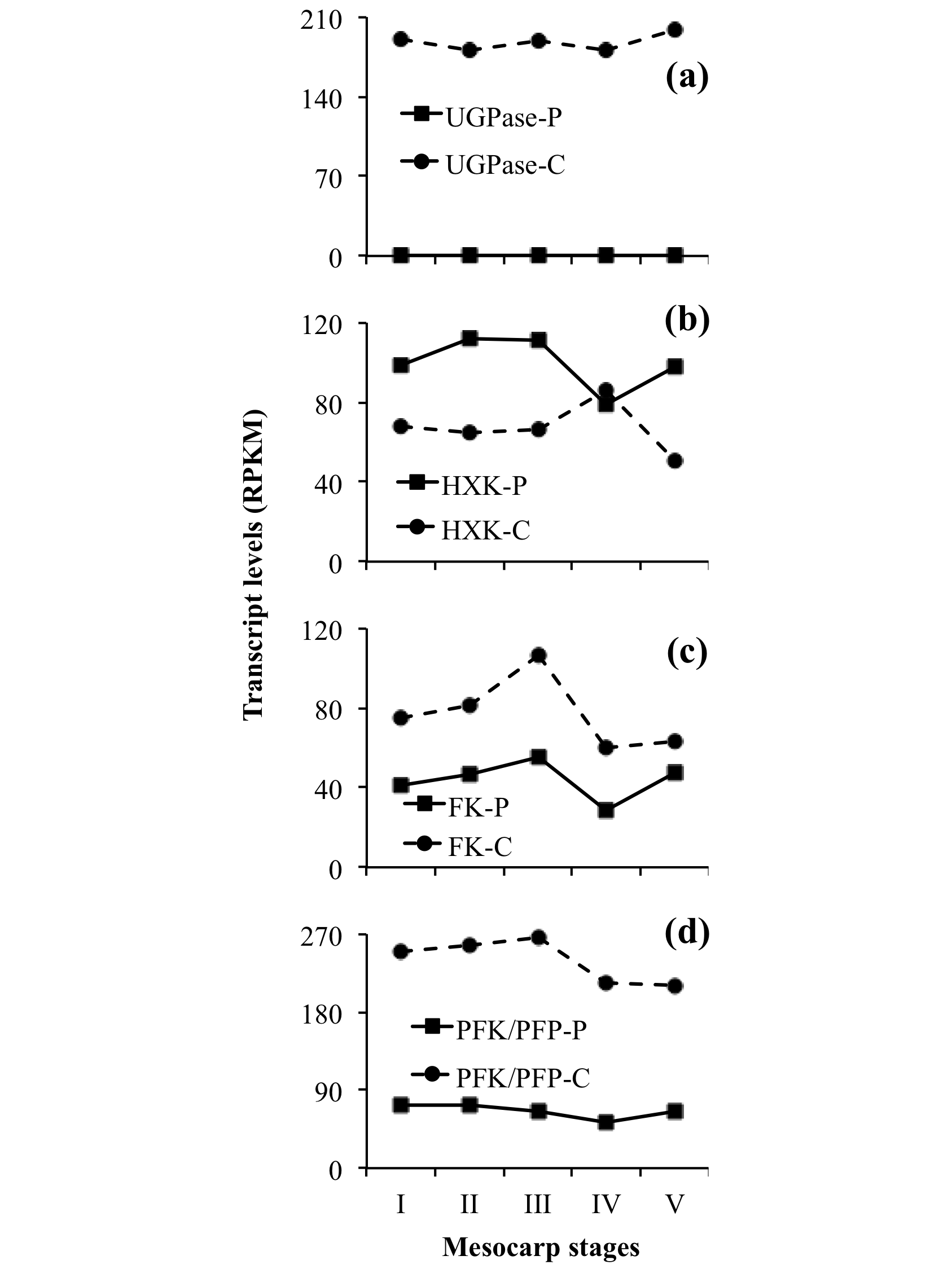

Supplement: Additional file 6: Figure S4. — Comparison of transcript levels for genes associated with early glycolysis in cytosol (C) and plastid (P). (a) UDP-glucose pyrophosphorylase (UGPase), (b) hexokinase (HXK), (c) fructokinase (FK) and (d) pyrophosphate-dependent phosphofructokinase (PFP) and ATP-dependent 6-phosphofructokinase (PFK). In plastids, PFK is predominant but PFP is absent. (TIFF 14823 kb) [file 12870_2015_586_MOESM6_ESM.tiff]

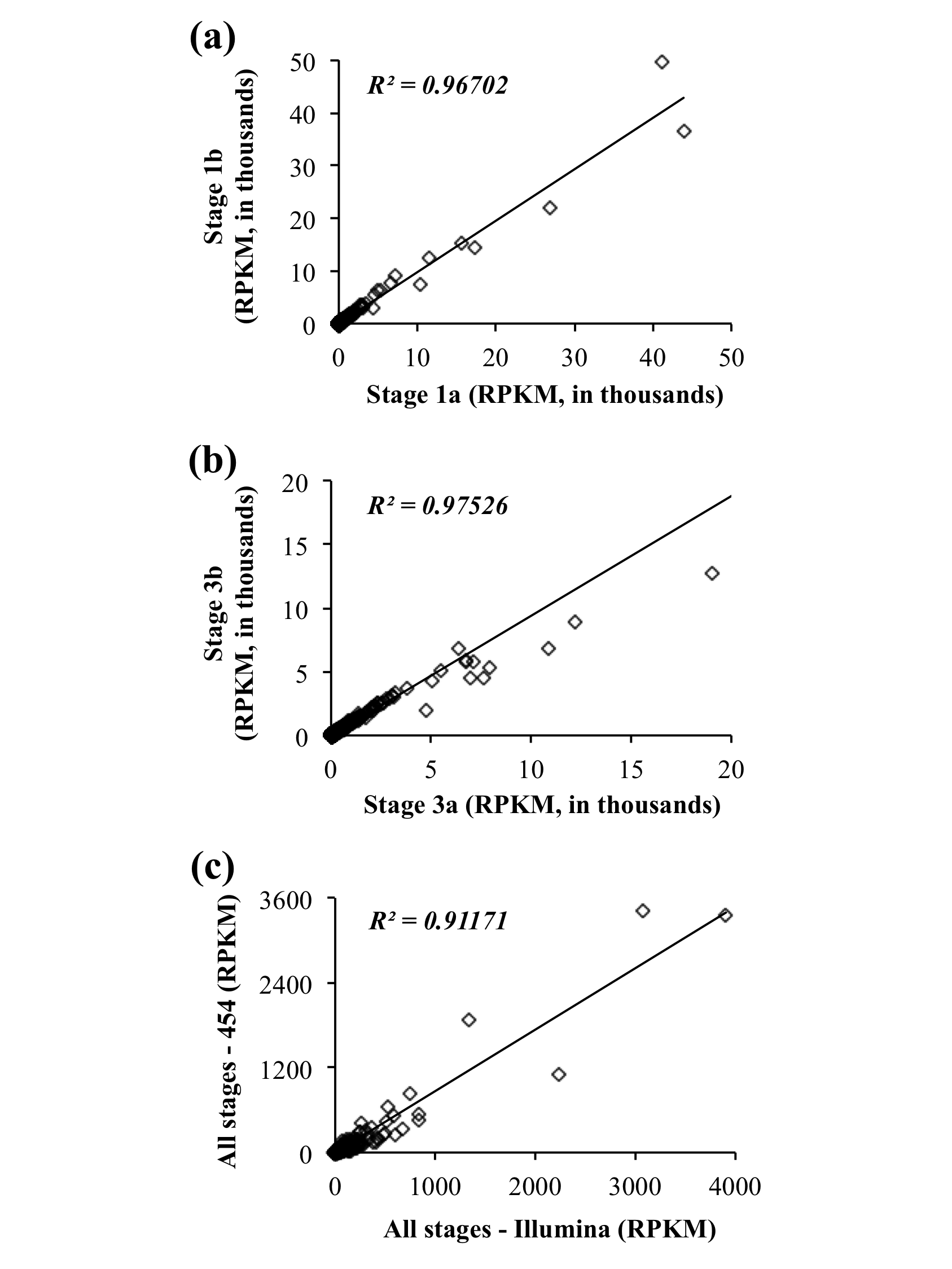

Supplement: Additional file 7: Figure S5. — Correlation analyses of RNA-seq data. (a) Correlation between the technical replicates of stage 1, and (b) and stage 3, obtained by Illumina sequencing. (c) Correlation between the 454 data and Illumina data obtained for ~ 250 gene orthologs used in the current study. (TIFF 14823 kb) [file 12870_2015_586_MOESM7_ESM.tiff]
